# Benign prostatic hyperplasia as a progressive disease: a guide to the risk factors and options for medical management

**DOI:** 10.1111/j.1742-1241.2008.01785.x

**Published:** 2008-07

**Authors:** M Emberton, E B Cornel, P F Bassi, R O Fourcade, J M F Gómez, R Castro

**Affiliations:** 1 Mark Emberton, Institute of Urology and Nephrology, University College LondonLondon, UK; 2 Department of Urology, Ziekenhuis Groop TwenteHengelo, The Netherlands; 3 Clinica Urologica, Università Cattolica del Sacro CuoreRome, Italy; 4 Service de Chirugie Urologique, Centre Hospitalier d’AuxerreAuxerre, France; 5 Servicio de Urología, Hospital Universitario Central de Asturias, Universidad de OviedoOviedo, Spain; 6 Clinical Development and Medical Affairs, GlaxoSmithKlineMadrid, Spain

## Abstract

Benign prostatic hyperplasia (BPH) is a complex disease that is progressive in many men. BPH is commonly associated with bothersome lower urinary tract symptoms; progressive disease can also result in complications such as acute urinary retention (AUR) and BPH-related surgery. It is therefore important to identify men at increased risk of BPH progression to optimise therapy. Several factors are associated with progression, including age and prostate volume (PV). Serum prostate-specific antigen level is closely correlated with PV, making it useful for determining the risk of BPH progression. Medical therapy is the most frequently used treatment for BPH. 5-alpha-reductase inhibitors impact the underlying disease and decrease PV; this results in improved symptoms, urinary flow and quality of life, and a reduced risk of AUR and BPH-related surgery. Alpha-blockers achieve rapid symptom relief but do not reduce the overall risk of AUR or BPH-related surgery, presumably because they have no effect on PV. Combination therapy provides greater and more durable benefits than either monotherapy and is a recommended option in treatment guidelines. The Combination of Avodart® and Tamsulosin (CombAT) study is currently evaluating the combination of dutasteride with tamsulosin over 4 years in a population of men at increased risk of BPH progression. A preplanned 2-year analysis has shown sustained symptom improvement with combination therapy, significantly greater than with either monotherapy. CombAT is also the first study to show benefit in improving BPH symptoms for combination therapy over the alpha-blocker, tamsulosin, from 9 months of treatment.

## Introduction

Benign prostatic hyperplasia (BPH) is a progressive disease that is commonly associated with bothersome lower urinary tract symptoms (LUTS) such as urinary frequency, urgency, nocturia, decreased and intermittent force of stream and the sensation of incomplete bladder emptying. The term ‘BPH’ actually refers to a histological condition, namely the presence of stromal-glandular hyperplasia within the prostate gland (1). The condition becomes clinically relevant if and when it is associated with bothersome LUTS; however, the relationship between BPH and LUTS is complex, because not all men with histological BPH will develop significant LUTS, while other men who do not have histological BPH will develop LUTS. Benign prostatic enlargement (BPE) is another component of the LUTS/BPH constellation (1). Reflecting the complex relationship between age-related changes in the prostate, not all men with histological BPH will develop BPE; in addition, not all men with LUTS will have concomitant BPE, and not all men with BPE will have bothersome LUTS. The final component of this complex relationship is bladder outlet obstruction (BOO). This results from a pressure gradient at the bladder neck/prostatic urethra and may lead to compression of the urethra, compromised urinary flow and deterioration of the upper urinary tract with renal failure (1). Yet again, not all men with BPH/BPE and LUTS will have BOO, and there are causes of BOO other than BPH/BPE (e.g. primary bladder neck sclerosis or a urethral stricture).

The causes of LUTS are multifactorial, although BPE secondary to BPH is a major contributing factor. The prevalence of LUTS in Europe varies with age, ranging from 14% for men in their fourth decade of life to > 40% for men in their sixth decade (2). Studies indicate little cultural variation in the prevalence of LUTS across Europe (3). Based on an overall prevalence of LUTS of 30%, approximately four million men aged > 40 years have LUTS in the UK alone (2). Furthermore, with elderly people constituting a greater proportion of the population, the prevalence of BPH and its impact on medical practice will increase.

Although bothersome LUTS are commonly the only determinant for a BPH diagnosis in clinical practice, simple investigations exist that can be highly effective in accurately diagnosing LUTS because of BPH. The European Association of Urology (EAU) guidelines recommend a series of initial evaluations for men with LUTS suggestive of bladder obstruction; these include taking a clinical history, using a validated questionnaire to assess symptoms, conducting a physical examination, creatinine measurement, urinalysis, flow rates, postvoid residual (PVR) volume and serum prostate-specific antigen (PSA) measurement (particularly when a diagnosis of prostatic carcinoma would affect the decision about which therapeutic option to use) (4). The initial evaluations recommended by the American Urological Association (AUA) are a clinical history, use of a validated questionnaire to assess symptoms, a physical examination, urinalysis and serum PSA measurement (5). A recent study demonstrated a high correlation between diagnoses using medical history, serum PSA, digital rectal examination (DRE) and International Prostate Symptom Score (IPSS) and those based on a full battery of tests including ultrasonography and uroflowmetry (6). Hence, initial investigations using simple diagnostic tools available in the primary care setting can offer a first diagnostic step in patients with suspected BPH, as well as a valid strategy to minimise delay in disease management and facilitate appropriate referral from primary to specialised care (6).

Our growing insight into the natural history of BPH and the physiological effects of medical interventions is increasing our understanding of how the tools available to us can guide therapeutic choices for individual patients. This review discusses current knowledge of the progressive nature of BPH and how the risk of BPH progression can be evaluated to guide therapeutic choices.

## BPH progression: definitions and prevalence

An expert review of published evidence regarding BPH as a progressive disease defined progression as worsening of symptoms, deterioration of urinary flow rate, increase in prostate volume (PV), and outcomes such as acute urinary retention (AUR) and the need for surgery either for AUR or symptoms (7). Clinical trials have included renal insufficiency and recurrent urinary tract infections as additional measures of BPH progression, although these outcomes were rarely observed (8). Both epidemiological studies and the placebo arms of randomised controlled trials provide strong evidence that BPH is a progressive condition in many men.

### Symptom progression

One of the largest and longest running longitudinal studies, the Olmsted County study conducted in the USA, recruited a sample of 2115 men aged 40–79 years. At 92 months’ follow-up, 31% of participants reported a ≥ 3-point increase in AUA Symptom Index (AUA-SI; identical to the seven symptom questions of the IPSS) score and the mean annual increase in AUA-SI was 0.34 points (9). In the placebo arm of the Medical Therapy of Prostatic Symptoms (MTOPS) study the rate of overall clinical progression (defined as an increase in AUA-SI of ≥ 4 points, AUR, urinary incontinence, renal insufficiency or recurrent urinary tract infection) was 17.4% over the 4-year duration of the study. About 78% of progression events took the form of deterioration in symptoms (8).

### Progression to AUR or BPH-related surgery

Although AUR and surgery are less common than overall symptomatic worsening, they are important progression events with financial, emotional and health-related consequences. In the Olmsted County study, the incidence of AUR in men with moderate-to-severe LUTS increased from 3.0/1000 person-years in those aged 40–49 years to 34.7/1000 person-years in those aged 70–79 years (10). By extrapolating data from this study, the overall risk of AUR has been estimated as 23% for an average 60-year-old man if he survives another 20 years (10). In the MTOPS study, 12% of progression events were AUR (8). AUR results in prostatectomy in 24–42% of men in the UK and USA, and even patients who avoid surgery through a successful trial without catheter are subsequently at high risk of requiring surgery within a year (11–13). Furthermore, longitudinal data from the Veteran's Affairs study in the USA has demonstrated that 36% of men with BPH randomised to watchful waiting switched to invasive therapy within 5 years of enrolment (14).

### Which progression events are of most concern to our patients?

The above data provide convincing evidence that BPH is a progressive disease in many men and that progression can take a variety of forms in individual patients. The most common complaint associated with BPH is bothersome LUTS. However, some patient surveys have reported that the risk of surgery is a greater concern for patients than other factors such as symptoms or even quality of life (15–17). In a survey of men treated with finasteride, 93% of respondents ranked reducing the need for surgery as very or extremely important and 88% said that reducing the risk of major urological complications was very or extremely important, while symptoms and quality of life were considered less important (16). Similarly, a survey of men with BPH in five European countries (PROBE) (17) found that 58% (68/117) and 56% (153/273) of patients were ‘fairly’ or ‘very’ concerned about the risk of developing AUR and requiring prostate-related surgery, respectively. Halving the risk of requiring surgery was ranked as a more important drug treatment outcome than rapid symptom relief by more than three-quarters of men in this study. This highlights the need for effective communication with patients so that treatment decisions are driven by an understanding of what concerns them most.

## Identifying men at risk of BPH progression

It is important for clinicians to determine which patients are at increased risk of disease progression in order to optimise therapy and offer a treatment approach that correlates with patient preferences. Numerous factors, such as age and PV, have been linked with the risk of BPH progression events (10,18,19). Other predictors include reduced urinary flow, increased symptom score and increased bother, which drive healthcare-seeking behaviour. Data from the placebo arm of the MTOPS trial demonstrated that men with a total PV ≥ 31 ml, PSA ≥ 1.6 ng/ml, *Q*_max_ < 10.6 ml/s, PVR volume ≥ 39 ml or age ≥ 62 years at baseline had a significantly increased risk of overall clinical progression of BPH (20).

Several studies have demonstrated a relationship between age and markers of BPH progression. In the Olmsted County study, moderate-to-severe urinary symptoms were recorded in 13% and 28% of men aged 40–49 years and > 70 years respectively (21). An increase in symptom severity with increasing age has also been reported in Asian men (22). As previously discussed, the incidence of AUR among men with moderate-to-severe symptoms in the Olmsted County study was shown to increase with increasing age (10). More recently, a study of men (*n* = 1859) with symptomatic BPH showed an increase in PV with increasing age, from a mean of 27.7 ml in men aged 40–49 years to 52.3 ml in men aged 70–80 years (23).

Prostate volume is perhaps the most extensively studied of the risk factors for BPH progression. Men with a PV of ≥ 30 ml are more likely to suffer moderate-to-severe symptoms (3.5-fold increase), decreased flow rates (2.5-fold increase), and AUR (three- to fourfold increase), compared with men with PV < 30 ml (24). An enlarged prostate is also predictive of the need for BPH-related surgery (10,19). Unfortunately, PV estimated by DRE is associated with considerable measurement error and can underestimate measured volume by as much as 55% (25,26). Transabdominal ultrasound can be used to assess PV, although there are limitations with this technique for imaging the prostate compared with the more invasive transrectal ultrasound (27). However, extensive data from the placebo arm of the Proscar Long-term Efficacy and Safety Study (PLESS) have demonstrated that PSA is a strong predictor of an enlarged prostate, or one that is likely to increase in size, as well as the risk of developing LUTS, poor urinary flow, AUR and/or BPH-related surgery (19). Although the precise relationship between PSA and prostate growth may vary from one individual to another (23,28), analysis of the data from PLESS showed that PSA thresholds for detecting a PV ≥ 30 ml were ≥ 1.3 ng/ml, ≥ 1.5 ng/ml and ≥ 1.7 ng/ml in men with BPH aged 50–59, 60–69 and 70–79 years, respectively (18). This relationship was confirmed by a retrospective analysis of 1859 Dutch patients in which 89% of those with a PSA ≥ 1.5 ng/ml had a PV > 30 ml (23). Studies also demonstrate a correlation between PSA and PV in Asian men (29,30).

Although it is important to note the limitations of extrapolating these findings to individual patients, the data suggest that the correlation between PSA and PV is close enough for serum PSA to be used to estimate the degree of prostatic enlargement. Indeed, PSA alone showed a sensitivity for predicting the progression of BPH comparable with the use of an algorithm and the five-variable model (encompassing the indices of serum PSA, symptom problems, peak urinary flow, urinary frequency ≤ 2 h and hesitancy) (31). The current EAU guidelines for the management of BPH reflect this by stating that PSA (as a proxy parameter for PV) can be used to evaluate the risks of either requiring surgery or developing AUR (4).

Serum PSA is easily measured in clinical practice and can therefore facilitate identification of those men most likely to suffer disease progression in BPH and to guide therapeutic decisions. Recent studies have shown that dynamic variables such as symptom score and PVR volume worsening also serve as good predictors of AUR in men with LUTS suggestive of BPH (32). Measurement of PVR volume is also recommended in the EAU guidelines (4), and this can be done routinely using transabdominal ultrasound.

## Medical therapy for BPH: how can we optimise the management of men with BPH?

Over the last decade, there has been a considerable decline in the popularity of surgery to manage symptoms associated with BPH, and medical therapy is now the most frequently used treatment option in clinical practice (33). Hence, patients with mild or moderate symptoms can usually be treated in a primary care setting, with more complicated cases referred to an urologist for evaluation and management (34).

Treatment of LUTS with plant extracts (phytotherapies) has a long tradition in countries such as France and Germany, and is also popular in other parts of the world (4). However, their mode of action is unclear and the clinical efficacy of these agents is largely unproven (35). Additional well-designed clinical studies are therefore needed before plant extracts can be recommended for the treatment of LUTS (4). Current EAU guidelines focus on alpha-blockers and 5-alpha-reductase inhibitors (5ARIs), as monotherapies or in combination, when recommending medical therapy for BPH (4).

### The role of alpha-blockers

#### Effects on symptom scores and symptomatic progression

The EAU guidelines include alpha-blockers as a treatment option for BPH where LUTS are bothersome and there is no absolute indication for surgery (4). They work by inhibiting alpha_1_-mediated sympathetic stimulation, and hence promote relaxation of prostatic smooth muscle and the bladder neck; additional sites of action include the bladder and central nervous system (36). Alpha-blockers have a rapid onset of action, with typical improvements in symptom scores of 30–45% after 3 months’ treatment (37–40). In the MTOPS study, doxazosin reduced the risk of symptomatic progression (defined as a four-point increase in AUA-SI) by 45% relative to placebo (8).

#### Effects on AUR and BPH-related surgery

Treatment with an alpha-blocker has not been shown to reduce the overall long-term risk of AUR or BPH-related surgery (8,41). In the MTOPS study, doxazosin delayed the time to AUR but did not significantly reduce the cumulative incidence at 4 years compared with placebo. Similarly, doxazosin did not significantly reduce the cumulative incidence of invasive therapy. In the Alfuzosin Long-Term Efficacy and Safety Study, alfuzosin did not reduce the risk of AUR compared with placebo; although a trend towards a lower incidence of BPH-related surgery was seen in the alfuzosin group, this did not reach statistical significance when compared with the placebo group (41). A logical explanation for this would be the observation that, to date, large-scale studies have not demonstrated a significant effect of alpha-blockers on PV. In fact, the MTOPS study showed that patients receiving doxazosin experienced a 24% increase in PV after an average follow-up of 4.5 years, identical to the increase observed in the placebo group (8).

Interestingly, data from a ‘real-life’ practice study suggest that men not responding to alfuzosin treatment (IPSS stable or worsening, and bother score > 3 under treatment) have a greater risk of AUR or BPH-related surgery. First-line treatment with alfuzosin might therefore help to select patients at risk of BPH progression and to optimise their management (32).

#### Tolerability

The adverse events (AEs) most commonly observed with alpha-blockers at a significantly higher frequency than with placebo are dizziness and postural hypotension, although there may be differences in the rates of such events between individual agents within the class (40). Alfuzosin and tamsulosin are better tolerated than terazosin and doxazosin, especially for cardiovascular AEs (40). Studies have demonstrated a higher incidence of abnormal ejaculation with tamsulosin compared with placebo (42–45).

### The role of 5-alpha-reductase inhibitors

**Figure 1:**
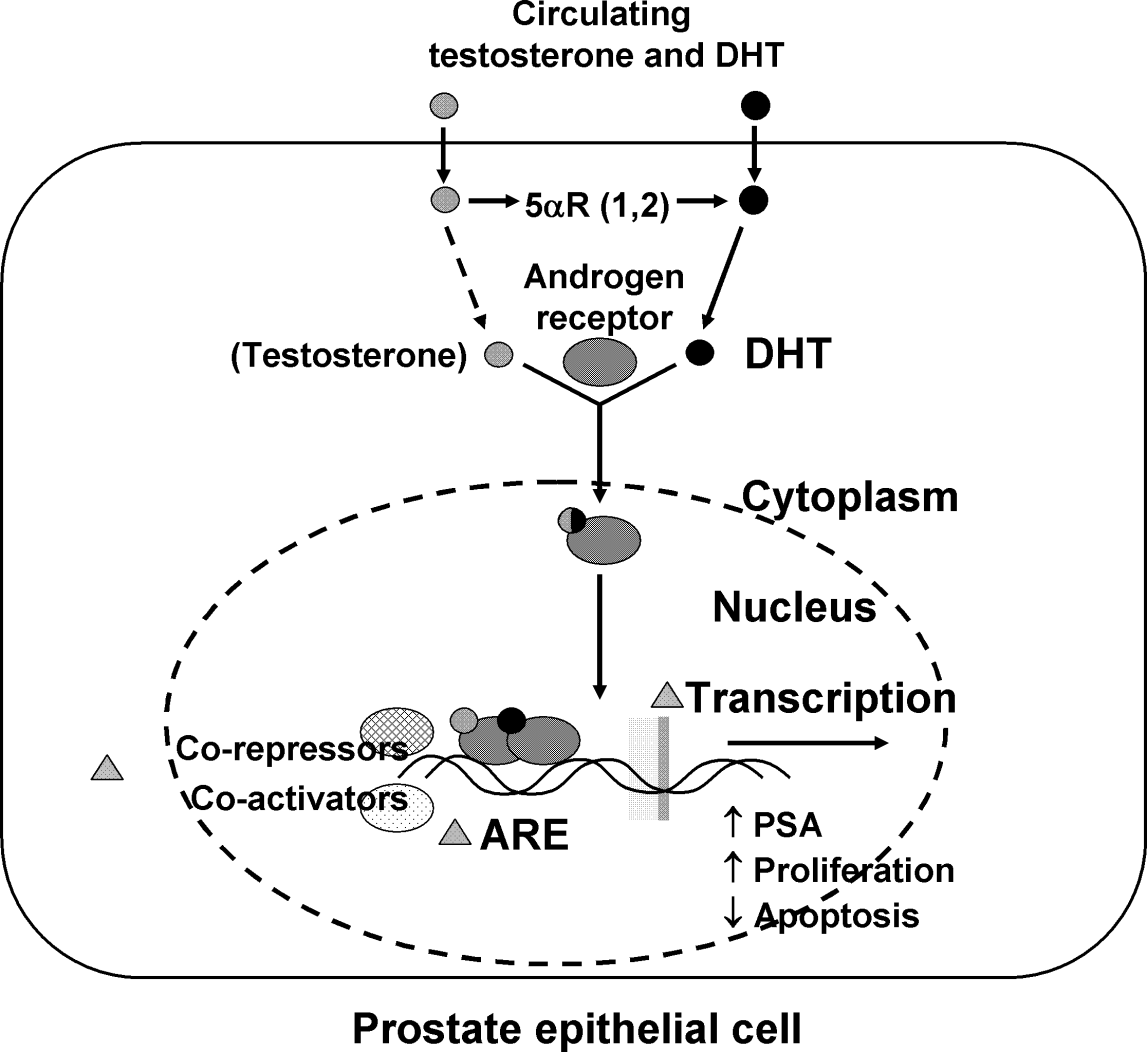
The role of 5-alpha-reductase (5αR) and dihydrotestosterone (DHT) in prostate growth

Current EAU guidelines for the management of BPH recommend 5ARIs for the treatment of bothersome LUTS in men with a PV > 30–40 ml, when there is no absolute indication for surgery (4). Others have proposed that 5ARIs should be considered as first-line medical treatment in men with symptomatic, progressive BPH as indicated by a PV ≥ 30 ml and/or PSA ≥ 1.5 ng/ml (46). 5ARIs prevent the conversion of testosterone to dihydrotestosterone (DHT), the primary androgen involved in prostate development (Figure 1). Two 5ARIs are available for the treatment of BPH, dutasteride and finasteride, which differ in their profile of 5AR inhibition. At therapeutic doses, finasteride inhibits only the type 2 isoenzyme of 5AR, while dutasteride is a dual inhibitor of both 5AR type 1 and type 2. Treatment with dutasteride results in a greater degree and consistency of DHT suppression compared with finasteride. In a study of men with BPH, the mean per cent decrease in DHT was 94.7 ± 3.3% with 0.5 mg dutasteride, significantly lower (p < 0.001) and with less variability than the 70.8 ± 18.3% suppression observed with 5 mg finasteride (47).

#### Effects on symptom scores and symptomatic progression

**Figure 2:**
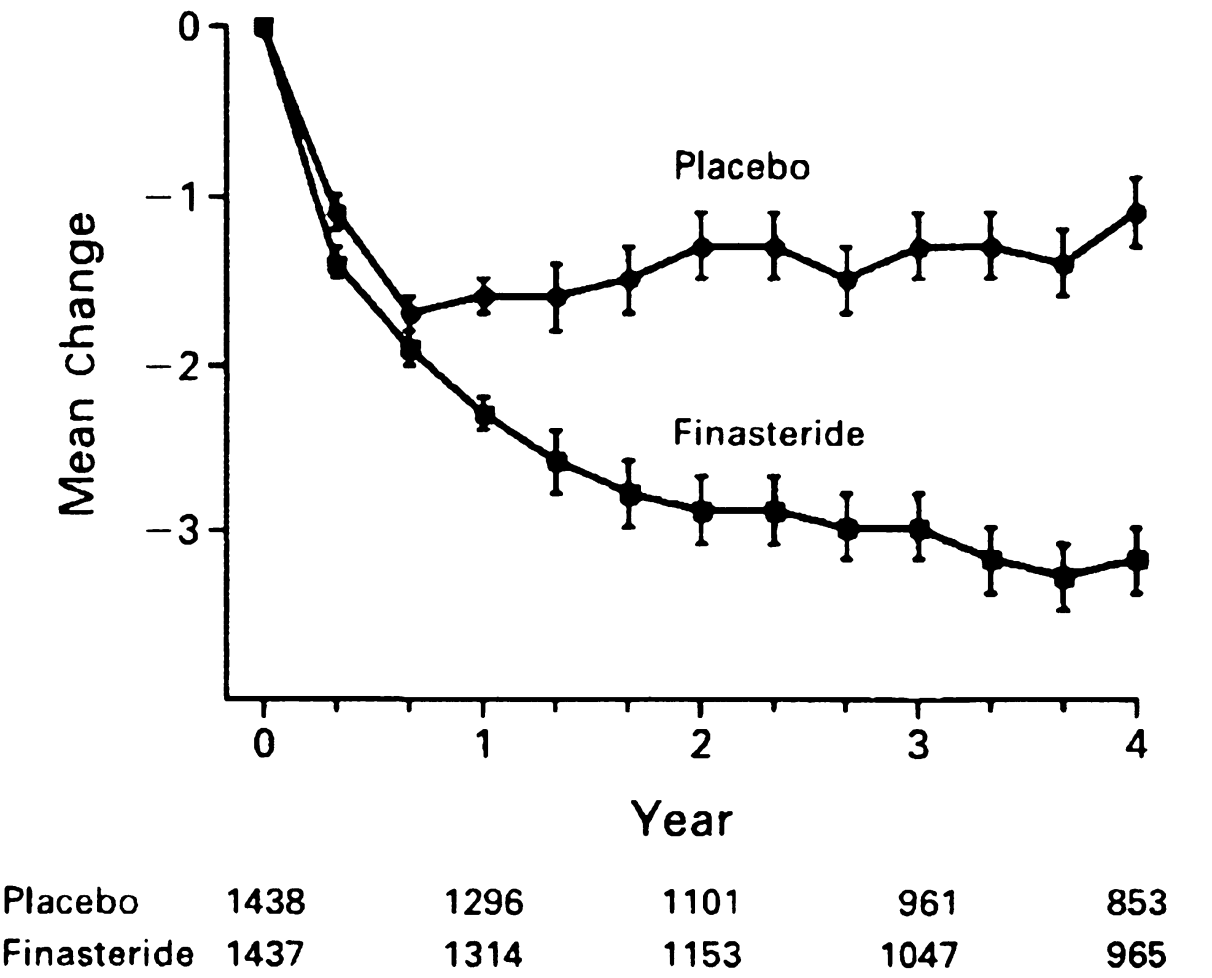
Effect of finasteride or placebo on symptom scores (on the Quasi-American Urological Association Symptom Scale) in the Proscar Long-term Efficacy and Safety Study. Reproduced from Ref. (18) with permission from the Massachusetts Medical Society. Values are mean (±SE) changes from baseline

In the 4-year PLESS study (*n* = 3040), finasteride treatment significantly improved symptom scores (2.6 points vs. 1.0 for placebo; p < 0.001) (18) (Figure 2). More recently, the MTOPS study showed that finasteride reduced the risk of symptomatic progression (defined as an increase in the AUA-SI ≥ 4 points) by 30% compared with placebo (p = 0.016). For the composite primary end-point of overall clinical progression (an increase in AUA-SI ≥ 4 points, AUR, urinary incontinence, renal insufficiency or recurrent urinary tract infection) finasteride reduced the risk by 34% relative to placebo (to 2.9 per 100 person-years; p = 0.002) (8).

**Figure 3:**
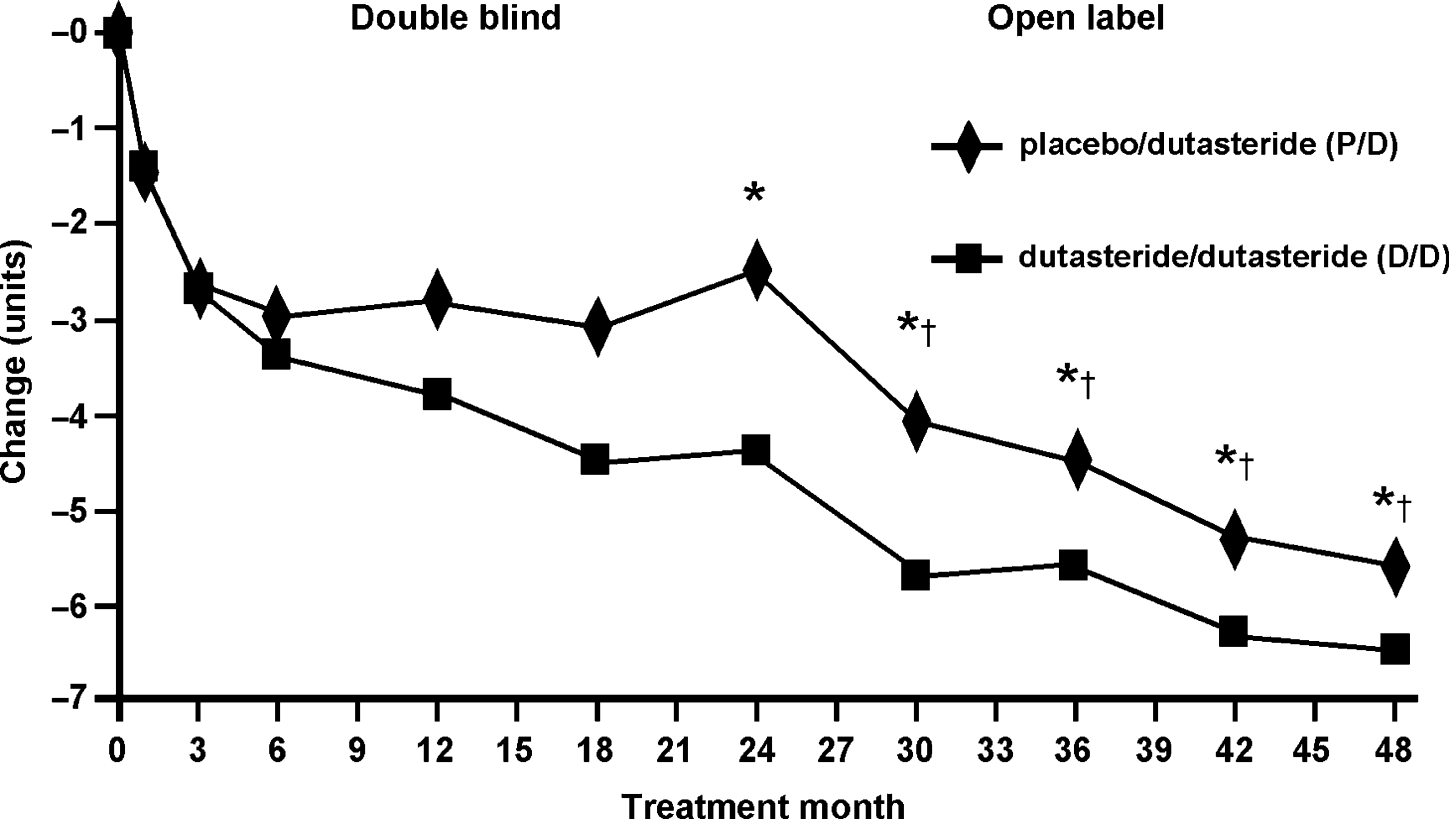
Mean change in American Urological Association Symptom Index scores from baseline over 48 months. Reproduced from Ref. (49) with permission from Elsevier. *p < 0.001 between treatment groups; †p < 0.001 for differences for both treatment groups from month 24

Pooled data from phase III studies with dutasteride (*n* = 4325) showed a 4.5-point improvement in symptoms at 2 years, compared with 2.3 points in the placebo group (p < 0.001) (48). Significant improvements in symptoms were observed from 6 months in the majority of patients, and from 3 months in some subjects. At the end of a 2-year open-label extension of these studies, improvements in symptom scores increased to 6.5 points (p < 0.001 vs. 2 years of dutasteride treatment) (Figure 3). Indeed, continuous improvement in symptoms was seen out to 4 years of treatment with dutasteride (49).

#### Effects on AUR and BPH-related surgery

In the PLESS study, after 4 years finasteride reduced the relative risk of AUR by 57% (7% of men receiving placebo and 3% of those receiving finasteride; p < 0.001) and surgery by 55% (10% of men receiving placebo and 5% of those treated with finasteride, p < 0.001) (18). A significant reduction in the risk of AUR and need for surgery in patients receiving finasteride monotherapy at 4.5 years (p < 0.001 vs. placebo) was also shown in the MTOPS study (8). Similarly, dutasteride studies have shown a reduced relative risk of AUR (57%) and a surgical intervention (48%) compared with placebo at 2 years (both p < 0.001 vs. placebo); this reduction was maintained to 4 years during open-label phases of the studies (48,49).

The reduced risk of AUR and BPH-related surgery with 5ARIs is probably mediated through their effects on PV. In the PLESS study, finasteride reduced PV by 18% compared with a 14% increase with placebo (mean difference 32%; p < 0.001), and in the MTOPS study a 19% reduction in PV was observed in patients receiving finasteride compared with a 24% increase in those receiving placebo (8,18). Phase III studies with dutasteride demonstrated a 26% reduction in PV relative to placebo, with significant reductions observed from 1 month after treatment initiation. PV reduction continued to study end-point at 2 years and was sustained for an additional 2 years in the open-label extension (48,49).

#### Tolerability

5-alpha-reductase inhibitors are generally well tolerated; sexual AEs are the most commonly reported side effects, although it is worth noting that there is a relatively high prevalence of sexual dysfunction in untreated men with BPH (50). Furthermore, studies have shown that the incidence of drug-related sexual AEs decreases during prolonged treatment (18,51). In the PLESS study, the rates of decreased libido and impotence were identical in finasteride and placebo groups after 2 years (18). Similarly, during 4 years of dutasteride treatment there was a trend towards a reduction in the rate of newly reported sexual AEs over time (51).

Although 5ARI therapy reduces serum PSA, this reduction is predictable and more importantly it does not jeopardise the diagnostic performance of PSA for detecting prostate cancer. Analyses of BPH clinical trials have shown that 5ARIs reduce serum PSA levels by approximately 50–60% after 6 months treatment. Hence, using doubled PSA values from 6 months onwards in 5ARI-treated men, and establishing a new baseline PSA level, preserves the clinical utility of the PSA test in prostate cancer detection irrespective of PSA level (52,53). Data from the PCPT show that treatment with finasteride may have actually enhanced the sensitivity of PSA for detecting all prostate cancers and high-grade disease because of preferential suppression of PSA related to BPH (54).

### The role of 5ARI/alpha-blocker combination therapy

It is clear from the available evidence that alpha-blockers offer rapid symptomatic relief without targeting the underlying disease process, while 5ARIs provide mid- and long-term symptom relief as well as a reduction in the risk of progression events such as AUR and BPH-related surgery. These complementary effects provide a sound rationale for 5ARI/alpha-blocker combination therapy, which provides greater and more durable benefits than either monotherapy and is a recommended treatment option in the EAU guidelines (4).

Initial studies, assessing finasteride in combination with terazosin, doxazosin or alfuzosin, failed to establish a benefit for combination therapy over placebo or alpha-blocker monotherapy in terms of improving LUTS or *Q*_max_ (55–57). However, these studies were limited by their short duration (6 months to 1 year), during which time a significant response to finasteride therapy was unlikely to occur. In contrast, the MTOPS study showed that the risk of long-term clinical progression was reduced by 66% with combination therapy (p < 0.001 vs. placebo) and to a greater extent than with either finasteride or doxazosin monotherapy (34% and 39% respectively) (8). All treatments resulted in a significant improvement in symptom scores, with combination therapy proving superior to either doxazosin (p = 0.006) or finasteride (p < 0.001) alone. The risks of AUR and need for BPH-related surgery over the 4-year study were significantly reduced with combination therapy and finasteride monotherapy (both p < 0.001) but not with doxazosin monotherapy (8). A recent analysis of the MTOPS data concluded that men with PV ≥ 25 ml and a PSA ≥ 1.5 ng/ml may benefit from combination therapy (58).

A large-scale study is currently examining the effects of the dual 5ARI dutasteride, both as monotherapy and in combination with tamsulosin, on symptoms and progression of BPH. The Combination of Avodart® and Tamsulosin (CombAT) study is a 4-year, international, multicentre study that differs from MTOPS in several important respects (59). While the MTOPS study inclusion criteria were largely related to symptoms, additional entry thresholds for PV (≥ 30 ml) and PSA (≥ 1.5 ng/ml) in the CombAT trial have been used to select patients who are at higher risk of disease progression. Furthermore, the independent assessment of symptom relief at 2 years and risk of AUR and surgery at 4 years in the CombAT trial, compared with the composite primary end-point of clinical progression in the MTOPS study, will allow a more transparent examination of which parameter is contributing to the overall treatment effect. This study therefore allows the assessment of those patients most likely to benefit from combination therapy, rather than evaluating the role of combination therapy across the entire spectrum of the disease.

**Figure 4:**
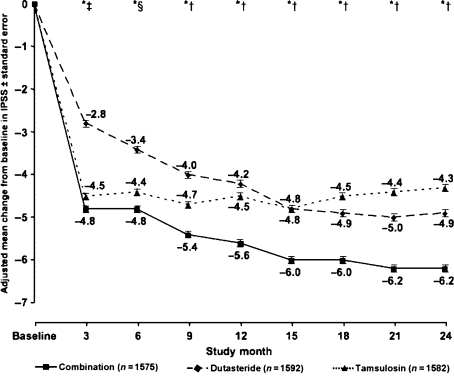
Change in International Prostate Symptom Score following treatment with dutasteride, tamsulosin or dutasteride/tamsulosin in combination. Reproduced from Ref. (60) with permission from Elsevier. *p < 0.001 for combination vs. dutasteride; †p < 0.001 for combination vs. tamsulosin; ‡p = 0.18 for combination vs. tamsulosin; §p = 0.032 for combination vs. tamsulosin

A preplanned analysis of CombAT at 2 years has shown sustained symptom improvement with combination therapy, and a significantly greater degree of improvement compared with either dutasteride or tamsulosin monotherapy (60,61). At month 24, reductions in the IPSS were −6.2 with combination therapy, −4.9 with dutasteride (p < 0.001 vs. combination therapy), and −4.3 with tamsulosin (p < 0.001 vs. combination therapy) (Figure 4). In addition, CombAT is the first study to show benefit in improving BPH symptoms for combination therapy over monotherapies in the first 12 months of treatment; a significant improvement vs. dutasteride was evident from month 3 and tamsulosin from month 9 (61). At month 24, the significance level in a *post hoc* analysis for dutasteride vs. tamsulosin was p = 0.013. Improvement in quality of life (as assessed by question 8 of the IPSS) was significantly greater for combination therapy (−1.4) than for either monotherapy at month 24 (change from baseline for both −1.1; p < 0.001 for both comparisons) (61). In addition, *Q*_max_ improvements from baseline were significantly better with combination therapy than with either monotherapy at all time points from month 6 to month 24 (each p ≤ 0.006); at month 24, mean changes from baseline were +2.4 ml/s with combination therapy, +1.9 ml/s with dutasteride and +0.9 ml/s with tamsulosin (61). Overall, a similar proportion of AEs were reported in each treatment group. Drug-related AEs were more common with combination therapy than with either monotherapy (particularly erectile dysfunction, ejaculatory disorders and decreased libido), although withdrawals due to AEs were low in all groups (61).

Studies have assessed the discontinuation of alpha-blocker and continuation of 5ARI therapy in men with symptomatic BPH receiving combination therapy (62,63). The results showed that symptom relief was maintained after alpha-blocker withdrawal in the majority of patients with moderate symptoms, whereas those with severe symptoms were more likely to experience worsening. However, the studies were of relatively short duration (9–12 months) and patient numbers were relatively small. There is now evidence from two large, randomised studies (MTOPS and CombAT) to demonstrate the benefits of long-term combination treatment over monotherapy for improving symptoms (8,61).

## Discussion and conclusions: how should the evidence guide treatment choices in BPH?

Benign prostatic hyperplasia is a chronic, complex disease that is progressive in many men. Although symptom deterioration is the most frequently occurring progression event, patients are often more concerned about progression to events such as AUR or BPH-related surgery. It is interesting to note that the risk of AUR is comparable with other conditions that are linked with ageing, such as diabetes, stroke and myocardial infarction, for which preventative approaches are commonplace. Given the significant pain, discomfort, economic and emotional burden associated with AUR and prostate surgery, it is important to consider therapeutic approaches that reduce the risk of such progression events while also achieving symptom relief. This approach responds to the needs of patients and is endorsed by the current EAU guidelines. Although alpha-blockers provide rapid symptom relief that is maintained in the long term, 5ARIs have been shown to provide continued symptom improvements (to 4 years with dutasteride) while also reducing the risk of progression to AUR or BPH-related surgery.

Men with enlarged prostates (> 30 ml) are at particularly high risk of disease progression and should receive the most effective management strategy available, derived from current guidelines and existing evidence. Although accurate PV measurement is difficult using DRE, elevated PSA is an accurate predictor of an enlarged prostate (once prostate cancer has been ruled out) and it can be used as a helpful tool to estimate PV. A PSA threshold of ≥ 1.5 ng/ml should be used to identify patients at high risk of BPH progression. However, serum PSA is also an important component in the detection of prostate cancer, and a concentration of > 4 ng/ml requires further evaluation and consideration of prostate biopsy. Dynamic variables such as symptom scores and PVR volume may also be helpful in predicting those men at risk of suffering AUR.

5-alpha-reductase inhibitors are the only therapy that alter the underlying disease process and prevent BPH disease progression. Although 5ARIs reduce serum PSA levels by approximately 50% after 6 months, they do not mask PSA changes; doubling the PSA measurement in treated men and establishing a new baseline preserves the utility of the test for detecting prostate cancer. In fact, recent data have shown that treatment with a 5ARI may enhance the sensitivity of the PSA test for prostate cancer detection (53).

**Figure 5:**
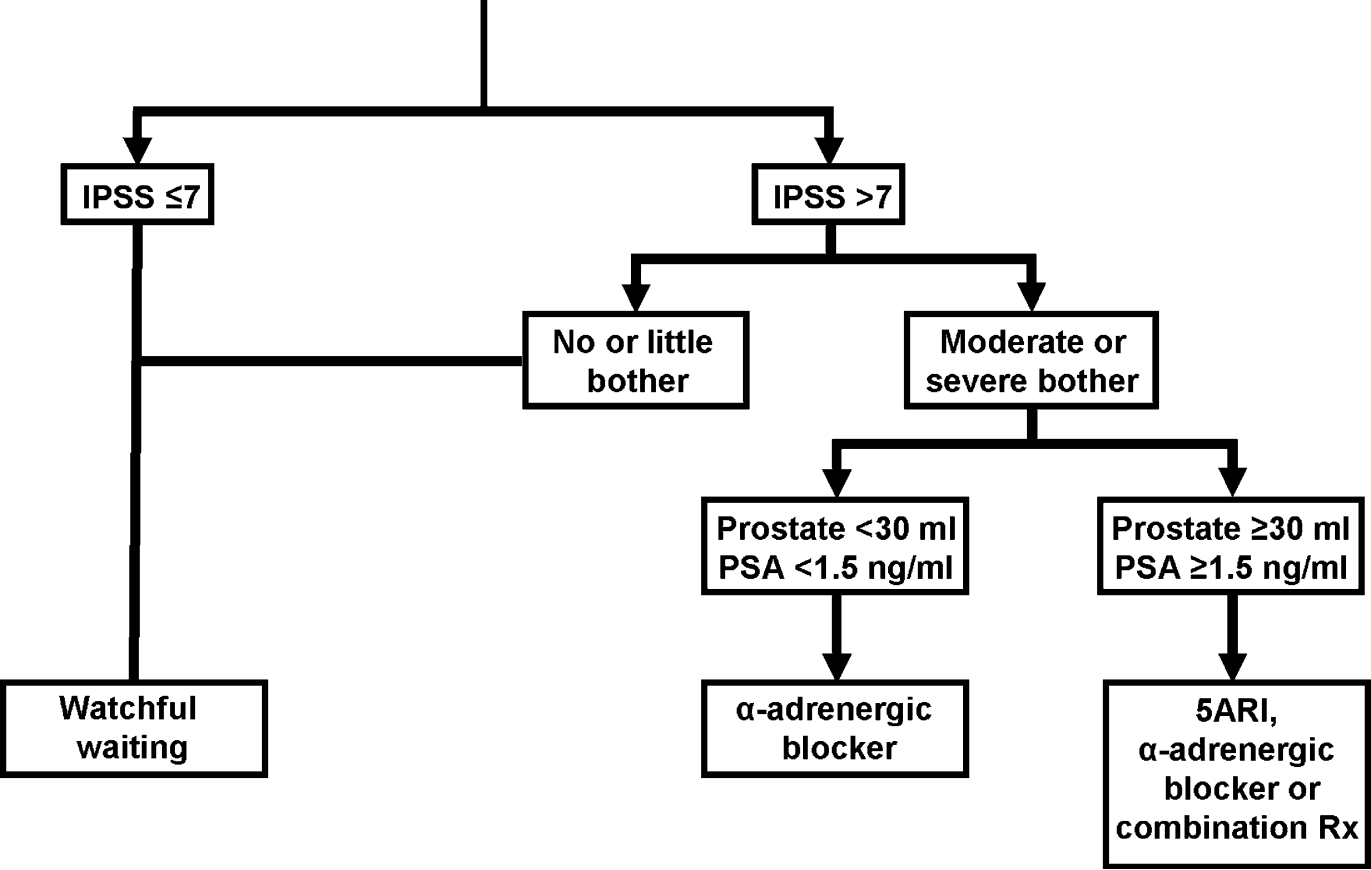
Proposed algorithm for medical therapy of benign prostatic hyperplasia. Adapted from Ref. (64) with permission from Macmillan Publishers Ltd

A proposed treatment algorithm, based on guideline recommendations and published evidence, is summarised in Figure 5 (64). In men with risk factors for BPH progression (including PV ≥ 30 ml and serum PSA ≥ 1.5 ng/ml) and an IPSS ≥ 7, 5ARI monotherapy is a recommended treatment option to minimise the risk of disease progression. For patients with an enlarged prostate and moderate-to-severe bothersome symptoms, who are unable to wait for the symptomatic benefit of a 5ARI, an alpha-blocker can be added to therapy. Ultimately, patients should be presented with a reasonable estimate of their baseline risk of progression along with the benefits and risks of medical therapy and the need for long-term treatment so that an informed decision can be made. Efficient and effective allocation of treatment according to severity and risk factors will result in fewer patients being treated with minimal benefits.

The CombAT study is underway to further examine the role of 5ARI/alpha-blocker combination therapy (dutasteride + tamsulosin) compared with monotherapy in the management of BPH. In comparison with the MTOPS study, men recruited into the CombAT study are deemed to be at higher risk of BPH progression, with baseline PV ≥ 30 ml and PSA levels of ≥ 1.5 ng/ml. A preplanned interim analysis of 2-year data from CombAT has shown sustained symptom improvement with combination therapy, with a greater degree of improvement compared with either dutasteride or tamsulosin monotherapy. In addition, CombAT is the first study to show benefit in improving BPH symptoms for combination therapy over the alpha-blocker, tamsulosin, in the first 9 months of treatment. The CombAT study represents the next major step in assessing the overall value of combination therapy in men with symptomatic BPH, and its findings will assist primary care physicians when making treatment decisions.
